# Multi-Physics Mesoscale Substructure Analysis on Stress Wave Measurement within CFST-PZT Coupling Models for Interface Debonding Detection

**DOI:** 10.3390/s22031039

**Published:** 2022-01-28

**Authors:** Jiang Wang, Bin Xu, Hongbing Chen, Hanbin Ge, Tianmin Zhou

**Affiliations:** 1 College of Civil Engineering, Huaqiao University, Xiamen 361021, China; 18011086007@hqu.edu.cn (J.W.); gehanbin@meijo-u.ac.jp (H.G.); 2 Key Laboratory for Intelligent Infrastructures and Monitoring of Fujian Province, Huaqiao University, Xiamen 361021, China; 3 College of Civil and Resource Engineering, University of Science and Technology Beijing, Beijing 100083, China; hongbingchen@ustb.edu.cn; 4 Department of Civil Engineering, Meijo University, Nagoya 468-8502, Japan; 5 Department of Civil and Environmental Engineering, University of Houston, Houston, TX 77204-4006, USA; zhoutm2011@gmail.com

**Keywords:** interface debonding detection, mesoscale concrete, multi-physics coupling models, embedded piezoelectric-lead-zirconate-titanate (PZT) sensor, concrete-filled steel tube (CFST), substructure

## Abstract

In recent years, the development of interface debonding defect detection methods for concrete-filled steel tubes (CFSTs) using stress wave measurement with piezoelectric-lead-zirconate-titanate (PZT) actuator and sensor has received significant attention. Because the concrete core in CFSTs is a heterogeneous material with randomness at the mesoscale, the size, position and distribution of aggregates unavoidably affect the stress wave propagation and the PZT sensor response. In this study, to efficiently investigate the influence of the mesoscale structure of the concrete core of CFSTs on the response of embedded PZT sensors, a multi-physics substructure model of CFST members coupled with a PZT actuator and a PZT sensor, where a single circular aggregate with different size and position and randomly distributed circular aggregates are considered, are established first. Then, multi-physics simulations on the effect of the local mesoscale structure of the concrete core on the response of the embedded PZT sensor excited by both a sinusoidal signal and sweep frequency signal are carried out. Moreover, corresponding multi-physics and mesoscale simulations on the embedded PZT sensor response of substructures with different interface debonding defects are also carried out for comparison. The amplitude and the wavelet packet energy of the embedded PZT sensor response of each mesoscale substructure are employed to distinguish the influence of the concrete core mesoscale structure and interface debonding defect on sensor measurement. The findings from the results with the multi-physics coupling substructure models are compared with those of the full CFST-PZT coupling models and the tested members of the previous studies to verify the rationality of the embedded PZT sensors measurement of the established substructure models. Results from this study show that the effect of interface debonding defect on the amplitude and the wavelet packet energy of the embedded PZT sensor measurement of the CFST members is dominant compared with the mesoscale heterogeneity and randomness of the concrete core.

## 1. Introduction

### 1.1. Problem Statement

As a typical concrete-steel composite structure, concrete-filled steel tube (CFST) members have been widely used as significant vertical and axial load-carrying members in long-span bridges and super high-rise buildings due to their advanced mechanical behavior under strong dynamic loadings, such as earthquakes, where the confinement effect of the steel tube on the concrete core plays a key role. However, the possible interface debonding defect between the concrete core and the steel tube has been a common concern due to shrinkage or apparent hydration heat. This is because the interface debonding defect weakens the confinement effect of the steel tube on the concrete core and finally leads to a negative effect on the load-carrying capacity, the stiffness and the ductility of CFST members. Therefore, it is particularly critical to develop effective interface debonding defect detection approaches for CFSTs [1].

In recent years, non-destructive testing (NDT) techniques, including the acoustic echo method, electromagnetic method, infrared thermal imaging method, ultrasonic method and the X-ray method have been proposed to detect different defects, including cracks and debonding between concrete and rebars in reinforced concrete (RC) structures [2,3,4,5,6]. Unfortunately, most existing methods do not work effectively for the interface debonding defect and voids detection for CFST members due to the electromagnetic shielding effect of the steel tube and the complexity of the internal structure of CFST members. Because the propagation of stress wave in steel tube and concrete core of CFST members is not affected by the electromagnetic shielding effect of steel tube, the detection approach for the interface debonding defect of CFST members with stress wave measurement using piezoelectric lead zirconate titanate (PZT) materials has received significant attention in recent years. With the advantages of the availability of different shapes, broad band response frequency, low price, and the ability of being employed as both an actuator and a sensor simultaneously, PZT patches have been widely employed in defect detection for concrete and RC structures and composite materials.

Investigation of the mechanism of defect detection for complex structures, including CFST members using PZT patch actuation and sensing technologies, is desired. Recently, numerical simulations on the stress wave propagation and the response of the PZT sensor embedded in or bonded on different structural members have been carried out to investigate the defect detection mechanism using stress wave measurements. The concrete core in most of the CFST numerical studies on stress wave propagation is usually modeled as a kind of homogeneous material [7,8]. In fact, concrete is a typical multi-phase and heterogeneous composite material, and the macroscale mechanical properties of concrete, including its Young’s modulus and strength, are closely related to its mesoscale compositions in terms of aggregates, mortar and the interfacial transition zone (ITZ) between them [9]. Therefore, it is desirable to further study the effect of the mesoscale structure of the concrete core in CFST members on the stress wave measurement of the PZT sensor to demonstrate the feasibility of defect detection for CFST members using stress wave measurement [10].

Even though a variety of mesoscale modeling methods for the purpose of numerically investigating the effect of the mesoscale structure of concrete on both local and global behavior of concrete materials and structures have been proposed [11,12,13,14,15,16,17,18], another challenging task for the high-frequency stress wave propagation simulation of a mesoscale CFST member model is the high computation cost and low computation efficiency as the very fine finite element model (FEM) and short integration time step are required. Therefore, it is necessary to develop an effective numerical method, such as a mesoscale substructure model for CFST members to efficiently explore the influence of the mesoscale structure of concrete, including the size, the position and the distribution of a single aggregate or aggregates between the PZT actuator and the embedded PZT sensor to demonstrate the feasibility of the defect detection approach for CFST members using a stress wave measurement where the concrete core should be considered as a heterogeneous material.

### 1.2. Literature Review on PZT Based Defect Detection for Engineering Structures

PZT materials have been used as either actuators or sensors for defect detection of different engineering structures. A low frequency bending piezoelectric actuator with integrated ultrasonic non-destructive evaluation (NDE) functionality was embedded in a composite laminate to detect damage using ultrasonic pulse excitation qualitatively [19]. In order to check the integrity of concrete structures, a PZT sensor was bonded to the concrete sample to evaluate the compactness of the concrete [20]. Considering the fragility of PZT materials, Ai et al. [21] developed PZT patches with impact resistance capability and used the developed PZT sensors to access the damage of concrete beams under an impact load. A new portable real-time Wireless impedance/Admittance Monitoring System (WiAMS) was developed for damage detection of shear-critical RC beams [22]. PZT sensors were arranged sequentially along a carbon fiber reinforced polymer (CFRP) to detect the initiation and propagation of cracks between the CFRP and concrete under static load [23]. For CFST members, a PZT-based interface debonding defect detection method was firstly proposed by Xu et al. [24], and the effectiveness of the proposed approach was demonstrated experimentally and numerically. Yan et al. [25] proposed a debonding detection method for a CFST column specimen using ultrasonic guided wave technology.

Perera et al. [26,27] used the frequency spectrum method to establish a finite element numerical model of FRP-reinforced RC beams and evaluated the damage of the models with varying degrees of damage with several PZT sensors. The FEM of the FRP-reinforced concrete beam was also established to verify the effectiveness of the electromechanical impedance (EMI) technology in monitoring FRP debonding [28]. Actually, the coupling effect between PZT and the host structures and the direct and inverse piezoelectric effects of PZT materials should be considered as a multi-physics problem when the mechanism of defect detection approaches for different engineering structures is investigated. Xu et al. [29,30,31,32,33,34] and Chen et al. [35] established multi-physics coupling FEMs for rectangular and circular CFST columns and numerically studied the stress wave propagation in the CFST members through transient dynamic analysis. The effect of different influencing parameters, including frequencies and amplitudes of excitation signals, the distance between the PZT actuator and the PZT sensor and different defect dimensions on stress wave propagation and the linear relationship between the PZT input signal and the output signal are discussed.

Most recently, in order to consider the mesoscale structure of the concrete core of CFST members, Xu et al. [36] first performed mesoscale simulations on the influence of circular aggregates distribution and the existence of ITZ on the stress wave propagation within a cross-section of CFST members where the concrete core has randomly distributed circular aggregates. The mesoscale simulation results show that, compared with the randomly distributed circular aggregates in the concrete core, the influence of the interface debonding defect on the embedded PZT sensor measurement is dominant. This study preliminarily confirmed the feasibility of the interface debonding defect detection method using the stress wave propagation measurement for rectangular CFST members where the concrete core has different distributed circular aggregates. In order to enhance the mesoscale simulation efficiency on stress wave propagation, Wang et al. [37] proposed a mesoscale homogenization method to improve the simulation efficiency of the stress wave field of mesoscale CFST models with differently distributed aggregates with different shapes. The results show that different mesoscale structures of the concrete core of healthy CFST models may lead to a certain difference in the output voltage signal of the embedded PZT sensor at a certain distance from the surface-mounted PZT actuator. However, the influence of the mesoscale structure between the PZT actuator and the embedded sensor on the measurement result was only investigated for the interface debonding defect detection on whole CFST members in the above study. Considering the fact that the stress wave propagation between the PZT actuator and sensor is affected by the local mesoscale structure of concrete, it is reasonable to investigate the influence of the mesoscale structure between the PZT actuator and sensor in a CFST substructure on the measurement result of an embedded PZT sensor using a substructure model.

### 1.3. Aim of This Study

In this paper, in order to efficiently investigate the influence of the mesoscale structure of the concrete core of CFST members on the output voltage response of the embedded PZT sensor and to compare it with that of the interface debonding defect, the multi-physics mesoscale simulation on the stress wave propagation of a substructure of CFST-PZT coupling models, considering different circular aggregate size, position, distribution and interface debonding defect, are carried out. The proposed mesoscale CFST-PZT coupling substructure model can not only study the influence of the mesoscale component, including aggregate dimension, position and distribution between the PZT actuator and embedded sensor on the response of the embedded sensor, but can also significantly improve the simulation efficiency.

In the mesoscale and multi-physics simulation on the stress wave propagation of the substructure, both sinusoidal excitation signals with different frequencies and sweep excitation signals are considered. The effect of the size, position of a single circular aggregate and the distribution of circular aggregates on the embedded PZT sensor response and the corresponding wavelet packet energy of healthy CFST members are investigated in detail. Then, the effect of the size, position of a circular aggregate and the distribution of circular aggregates on the embedded PZT sensor response and the corresponding wavelet packet energy of the corresponding CFST members with interface debonding defects are demonstrated and compared. Finally, the effect of the mesoscale structure of the substructure on the response of embedded PZT sensors is compared with the interface debonding defect and the finding from the numerical results with the substructure proposed in this study is compared with that of the previous experimental study by the authors. The rationality of the proposed mesoscale substructure model for stress wave propagation is demonstrated. Mesoscale substructure simulation results show that the mesoscale structure of the concrete core has a certain effect on the embedded PZT sensor response, but the effect of the interface debonding defect on the embedded PZT sensor measurement is dominant. The results imply that the interface debonding detection approach with stress wave measurement for CFST members is reasonable even though the concrete core is a heterogeneous material.

## 2. Control Equations for Multi-Physics Stress Wave Propagation of a Substructure of CFST-PZT Coupling Systems

### 2.1. Control Equations for Stress Wave Propagation

The control equations for elastic stress wave propagation simulation in a solid medium excited by a PZT actuator are shown in Equations (1)–(5) [29,38,39,40].

(1) $$\rho \frac{\partial^{2}u}{\partial t^{2}}=\nabla \cdot S+F_{V}$$

(2) $$S=S_{a}+C:\varepsilon_{e}$$

(3) $$\varepsilon_{e}=\varepsilon -\varepsilon_{0}$$

(4) $$\varepsilon =\frac{1}{2}\left[{\left(\nabla_{u}\right)}^{T}+\left(\nabla_{u}\right)\right]$$

(5) $$\nabla ={\left(\frac{\partial }{\partial x}+\frac{\partial }{\partial y}+\frac{\partial }{\partial z}\right)}^{T}$$

where ρ is the density of material, u represents the displacement vector, *t* is the time variable, ∇ is the gradient operator, S represents the stress tensor, $F_{V}$ stands for the external load vector, $S_{a}$ is the initial stress, C denotes elastic matrix, and $\varepsilon_{e}$ is the difference between strain ε and initial strain $\varepsilon_{0}$.

### 2.2. Control Equations of PZT Material in Solid and Static Electricity

The direct and inverse piezoelectric effects of the PZT sensor and actuator are considered in the coupling model composed of PZT patches and CFST members. The mechanical balance equation of PZT patches is consistent with the force balance equation of conventional solid materials except for the stress tensor shown in Equation (6).

(6) $$S_{P}=S_{Pa}+C_{PE}:\varepsilon_{Pe}-e_{P}^{T}E_{P}$$

where $S_{P}$ represents the stress tensor, $S_{Pa}$ is the initial stress, $C_{PE}$ stands for the inverse of the flexible matrix, $\varepsilon_{Pe}$ is the difference between the strain and initial strain, $e_{P}$ represents the piezoelectric stress constant matrix, and $E_{P}$ is the electric field strength.

The charge conservation equations of the PZT material in the electrostatic effect are shown in Equations (7)–(11) [29,38,39,40].

(7) $$\nabla \cdot D=\rho_{v}$$

(8) $$D=D_{r}+e_{P}\varepsilon_{Pe}+\varepsilon_{0,\;va}\varepsilon_{rs}E_{P}$$

(9) $$e_{P}=dC_{PE}$$

(10) $$\varepsilon_{rs}=\varepsilon_{rT}-C_{PE}d^{T}/\varepsilon_{0,\;va}$$

(11) $$E_{P}=-\nabla v$$

where D stands for the electric displacement, $\rho_{v}$ denotes the charge density, $D_{r}$ is the residual electric displacement, $\varepsilon_{0,\;va}$ and $\varepsilon_{rs}$ stands for dielectric constant in a vacuum and is the relative dielectric constant, d. refers to the coupling matrix, $\varepsilon_{rT}$ represents relative dielectric constant and V is the voltage.

### 2.3. Boundary Conditions Setting for a Substructure of the CFST-PZT Coupling Models

Different from the previous multi-physics mesoscale simulation studies by Xu et al. [35,36], in this study, a substructure between the PZT actuator and sensor coupled with the PZT actuator and sensor is investigated. The mechanical and electrical boundary for both the PZT actuator and sensor is identical to that described in the previous studies performed by Xu et al. In addition, the low reflection boundary along the three sides of the substructure is used.

The low reflection boundary condition used for the substructure of the CFST member coupled with the PZT actuator and sensor meets the following Equations (12) and (13) [29,30,31].

(12) $$\sigma \cdot n=-d_{im}\frac{\partial u}{\partial t}$$

(13) $$d_{im}=d_{im}\left(K,c_{s},c_{p}\;\right)$$

where σ is the stress matrix and n is the normal vectors of boundary, $d_{im}$ stands for a function where K denotes density, $c_{s}$ represents the shear wave velocity and $c_{p}$ is the longitudinal wave velocity.

## 3. Multi-Physics Mesoscale Modeling for a Substructure of Coupling CFST-PZT Systems

### 3.1. Multi-Physics Mesoscale Substructure Coupling Model

**Figure 1:**
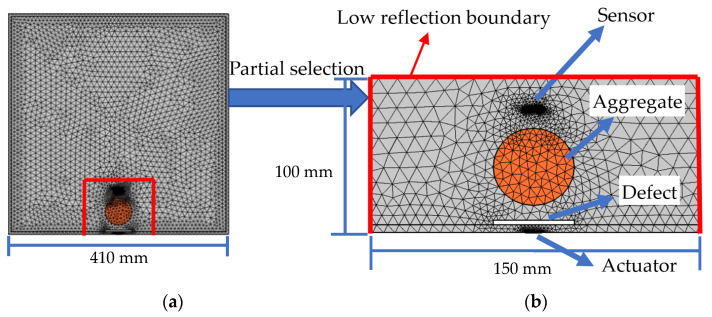
Cross-section and the FEM mesh of a CFST member with PZT patches as actuator and sensor: (**a**) whole model; (**b**) substructure model.

In this study, a mesoscale substructure, including a PZT actuator and a PZT sensor as shown in Figure 1b from a mesoscale rectangular CFST-PZT coupling model as shown in Figure 1a is considered and the output voltage response of the embedded PZT sensor in the mesoscale substructures is numerically simulated.

In order to consider the influence of the size and the position of circular aggregates on the stress wave propagation in the mesoscale substructure, a single circular aggregate with different dimensions and at different locations is considered in the mesoscale model. For comparison, an interface debonding defect is also considered in each mesoscale substructure model. Three surrounding boundaries of each mesoscale substructure are modeled with a low reflection boundary to limit the stress wave reflection on the boundaries for the purpose of mimicking the stress wave propagation within the whole cross-section of the CFST member.

The dimension of the whole rectangular CFST member is 410 mm × 410 mm, and the thickness of the steel tube is 5 mm. The dimension of the concrete core is 400 mm × 400 mm. The dimension of the mesoscale substructure is 150 mm × 100 mm. The length and the thickness of the employed PZT actuator and sensor are 10 mm and 0.3 mm, respectively. The PZT actuator is attached to the outer surface of the steel tube, and the PZT sensor is embedded in the concrete core to measure the stress wave. The distance between the PZT actuator and the PZT sensor is 80 mm, as shown in Figure 1b.

In order to investigate the influence of the mesoscale substructure of the concrete core of CFST members on the output voltage signal and compare it with that of the interface debonding defect, the following parameters, including the size, the lateral and longitudinal position of a single circular aggregate shown in Table 1 are considered, where the origin of the lateral and longitudinal position of the circular aggregate is defined as the midpoint of the line connecting the center points of the PZT actuator and sensor. Finally, three substructure models with different distributions of the circular aggregates taken from three mesoscale CFST models are also established to further investigate the effect of the heterogeneity and randomness of the mesoscale substructure of CFST members on PZT sensor measurement in the following section.

### 3.2. Material Properties of the Mesoscale Substructure Model

The mesoscale CFST-PZT coupling substructure models are composed of circular aggregates, mortar, and a steel tube, and their material parameters are shown in Table 2 [36].

### 3.3. Meshing of the Mesoscale Coupling Substructure

In order to accurately simulate the response of an embedded PZT sensor in the mesoscale substructure of each CFST-PZT coupling model, meshing with a suitable element dimension is critical. The maximum dimension of the elements should satisfy the following equation.

(14) $$h_{max}\leq \frac{v}{5f}$$

where $h_{max}$ is the maximum dimension of the element, v represents the wave speed and f is the frequency of excitation signal.

Here, three sinusoidal signals with different frequencies including 10 kHz, 20 kHz and 30 kHz are considered. The input voltage amplitude of the excitation signal is 10 V. The equation of the employed excitation signal is shown as follows.

(15) $$V_{t}=V_{0}\sin\left(2\pi ft\right)$$

where $V_{t}$ and $V_{0}$ refer to the excitation voltage signal and its amplitude, t is the time instant.

The element number of each mesoscale coupling substructure model shown in Figure 1b corresponding to different excitation frequencies is shown in Table 3.

## 4. Aggregate Effect on Steady Output Voltage Signal of the Embedded PZT Sensor of the Substructure without Debonding under Sinusoidal Signal

The response of the embedded PZT sensor of each mesoscale coupling substructure is numerically simulated when the size, lateral and position of a single circular aggregate and the distribution of circular aggregates in the substructure are considered.

### 4.1. Effect of the Size of a Single Circular Aggregate

**Figure 2:**
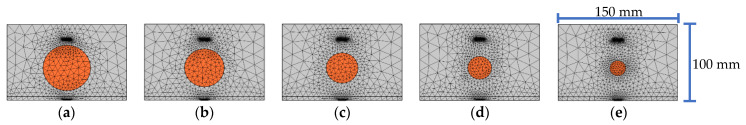
Mesoscale coupling substrucures with different size of a single circular aggregate: (**a**) 60 mm; (**b**) 50 mm; (**c**) 40 mm; (**d**) 30 mm; (**e**) 20 mm.

In order to investigate the effect of the size of a single circular aggregate on the response of the embedded PZT sensor in mesoscale coupling substructures, five mesoscale substructure coupling models with different circular aggregate sizes, as shown in Figure 2, are established. The center of the aggregate is identical and located at the midpoint of the line connecting the center points of the PZT actuator and sensor.

**Figure 3:**
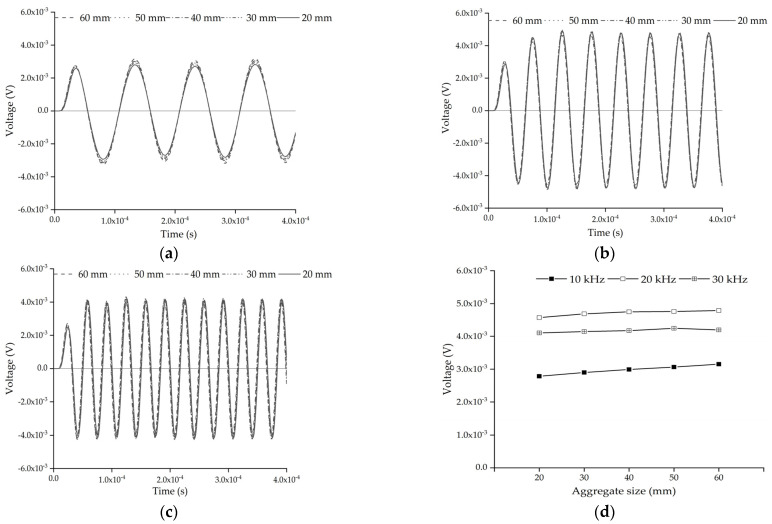
Response of the embedded PZT sensors in mesoscale coupling substructures with different circular aggregate sizes under sinusoidal signal: (**a**) 10 kHz; (**b**) 20 kHz; (**c**) 30 kHz; (**d**) the comparison of steady amplitudes.

Figure 3a–c shows the comparison of the time histories of the response of the embedded PZT sensor under different sinusoidal excitation signals when the size of the single circular aggregate is different. It can be seen from Figure 3a–c that the time history of the response of the embedded PZT sensor of each substructure with different circular aggregate sizes does not have an obvious difference under an identical continuous sinusoidal signal excitation. The comparison of steady output voltage amplitudes of the embedded PZT sensor in each substructure is shown in Figure 3d under the excitation of different frequencies. It can be found that when the frequency of the sinusoidal signal is 10 kHz, the output voltage amplitude of the PZT sensor increases slightly with the increase of aggregate size. When the sinusoidal signal frequency is less than or close to 20 kHz, the difference between the voltage amplitudes is minimal even though the aggregate has different diameters within the range of 20 mm–60 mm. For the five mesoscale substructure coupling models shown in Figure 2, the centers of the PZT actuator, the aggregate, and the PZT sensor are in a straight line. The response of the embedded PZT sensor of the model with a larger aggregate size is greater than that of others because the stress wave attenuates less in the aggregates than in the mortar.

### 4.2. Effect of the Lateral Position of a Single Circular Aggregate

**Figure 4:**
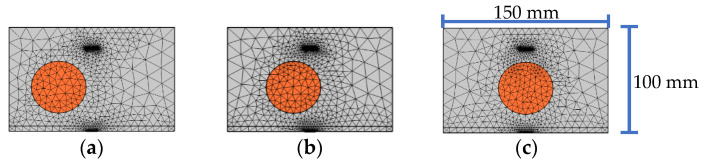
Samples of substructure with different lateral position of a single circular aggregate: (**a**) −30 mm; (**b**) −15 mm; (**c**) 0 mm.

**Figure 5:**
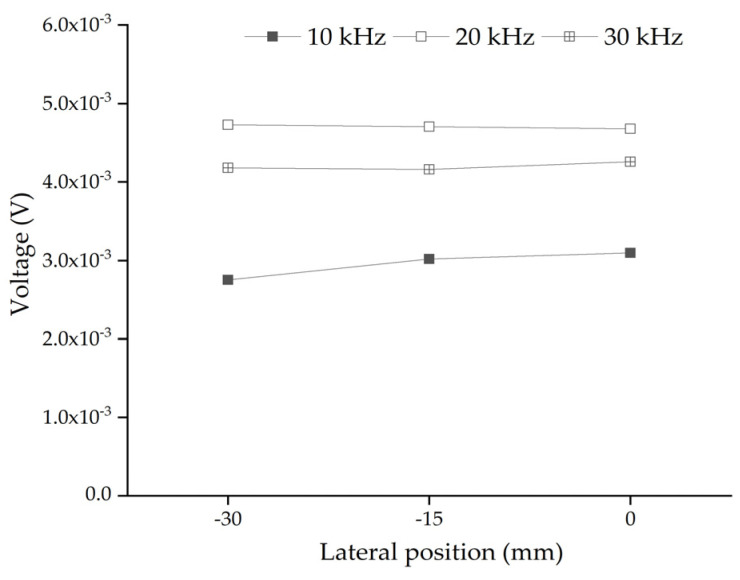
The comparison of the response amplitudes of embedded PZT sensors in substructures with different lateral positions under continuous sinusoidal signals with different frequencies.

In this section, the influence of the lateral position of a single circular aggregate between the PZT actuator and sensor on the response of the embedded PZT sensor is studied. Here, the size of the single circular aggregate is 50 mm. Figure 4 shows the mesoscale substructure coupling model with a circular aggregate relative to the PZT patches at different lateral positions. The response amplitude of the embedded PZT sensor under the continuous sinusoidal excitation signal at different frequencies is also shown in Figure 5.

Figure 5 shows the comparison of the steady amplitudes of the embedded PZT sensors in each substructure with a single circular aggregate at different lateral positions under sinusoidal excitation signals with different frequencies. It can be seen from Figure 5 that when the lateral position of the single circular aggregate is 0 mm, the steady amplitude of the response of the embedded PZT sensor is the largest. This result can also be explained from the difference in the stress wave attenuation in the aggregate and mortar. For the specimen shown in Figure 4a, more stress wave attenuates in mortar when it propagates from the actuator to the sensor. However, for the specimen shown in Figure 4c, less attenuation occurs when stress wave propagates from the actuator to the sensor due to the existence of the aggregate at the center between the actuator and the sensor.

When the frequency of the excitation signal is less than or close to 20 kHz, the steady amplitude of the response of the embedded PZT sensor does not obviously change with the circular aggregate lateral position. When the frequency of the excitation signal is close to 10 kHz, the steady amplitude of the embedded PZT sensor response has a certain variation due to the circular aggregate lateral position change. Since the lateral position of the single aggregate leads to differences in the output voltage response of the embedded PZT sensor when the frequency is close to 10 kHz, the frequency greater than or equal to 20 kHz is a better choice to limit the effect of the lateral position of the signal aggregate on the response of the embedded PZT sensor.

### 4.3. Effect of the Longitudinal Position of a Single Circular Aggregate

**Figure 6:**
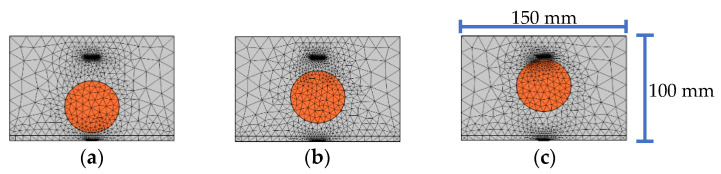
The samples of substructure models with different aggregate longitudinal positions: (**a**) −100 mm; (**b**) 0 mm; (**c**) 100 mm.

In order to investigate the effect of the longitudinal position of a single circular aggregate on the embedded PZT sensor response, three mesoscale coupling models considering different longitudinal positions of the single circular aggregate, as shown in Figure 6, are considered. The circular aggregate shown in Figure 6b is located in the middle of the PZT actuator and sensor, and the circular aggregate shown in Figure 6a,c is 100 mm below and above the middle point between the PZT actuator and sensor, respectively.

**Figure 7:**
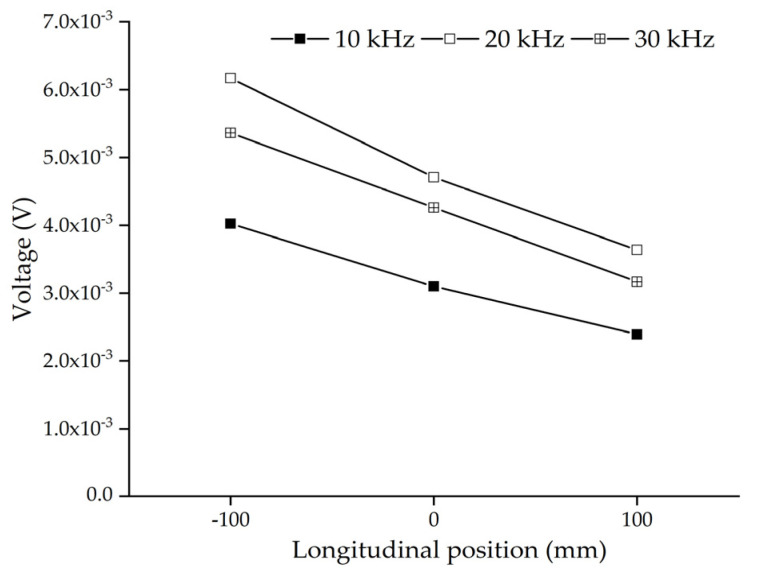
The comparison of steady amplitudes of the responses of the embedded PZT sensors in the mesoscale substructure coupling models with different longitudinal positions of the circular aggregate at different excitation frequencies.

Figure 7 shows the comparison of steady amplitudes of the response of the embedded PZT sensors in three mesoscale substructure coupling models under different frequencies. It can be seen from Figure 7 that the steady amplitude of the embedded PZT sensor response becomes smaller when the aggregate is closer to the PZT sensor, no matter which frequency is used. This observation can also be explained by the different stress wave attenuation in the aggregate and mortar and the wave field from the PZT actuator as a point source. When comparing the stress wave propagation in Figure 6a,c, more stress attenuation occurs in the mortar close to the PZT actuator in Figure 6c and less stress wave attenuation occurs in the aggregate close to the PZT actuator in Figure 6a. Therefore, the response of the PZT sensor in Figure 6c is less than that in Figure 6a. Moreover, the stress wave attenuation in the region close to the PZT actuator is dominant because the PZT actuator is a point source. Therefore, the steady amplitude of the embedded PZT sensors at a certain frequency changes obviously due to different longitudinal positions of the circular aggregate.

Comparing the results shown in Figure 7 with those shown in Figure 5, it can be concluded that the longitudinal position of the circular aggregate has a greater influence on the steady amplitude of the embedded PZT sensor than that of the lateral position of the circular aggregate in the healthy mesoscale CFST-PZT coupling substructure.

### 4.4. Effect of Circular Aggregates Distribution of the Substructure

**Figure 8:**
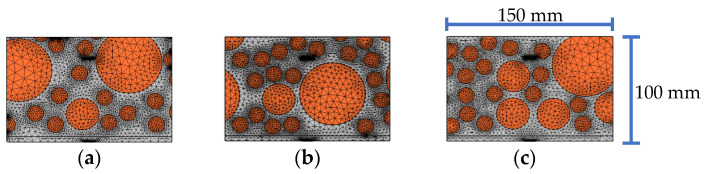
The samples of substructure models with different circular aggregates distribution for substructures: (**a**) sample 1; (**b**) sample 2; (**c**) sample 3.

In order to consider the influence of the randomness and heterogeneity of the mesoscale CFST-PZT substructure coupling model on the response of the embedded PZT sensor, three substructure models taken from three CFST-PZT coupling systems with different three-grade circular aggregates distributions are investigated. The three mesoscale substructure models with their FEM mesh are shown in Figure 8. It should be mentioned here that each mesoscale substructure coupling model is taken from a global mesoscale model with different three-grade circular aggregates distribution, but the numbers of the aggregates of the three grades of each global mesoscale are identical, which ensures that the macroscale mechanical parameters of each global mesoscale model are identical. The response of the embedded PZT sensor in the three mesoscale substructure coupling models under a sinusoidal excitation signal with different frequencies is simulated.

**Figure 9:**
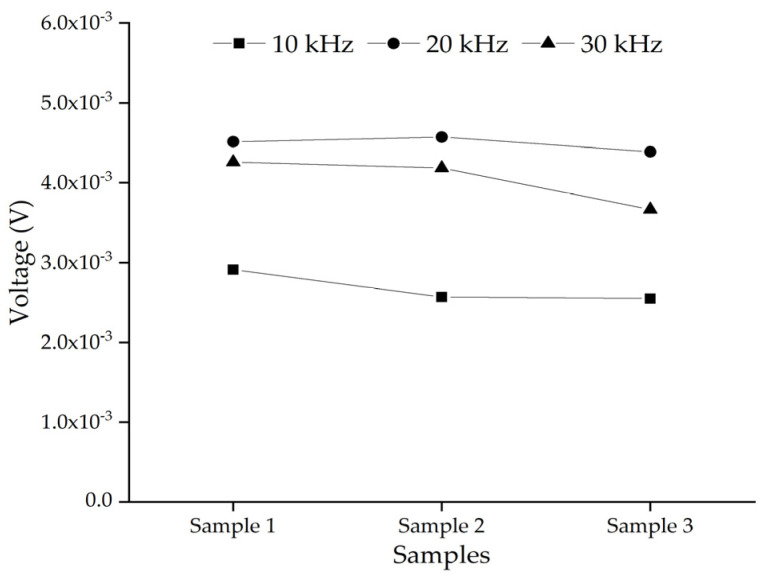
The comparison of the steady amplitude of the response of the embedded PZT sensors in substructure models considering different aggregates distribution under different excitation frequencies.

Figure 9 shows the comparison of the steady amplitude of the response of the embedded PZT sensors in the three mesoscale substructure coupling models when the randomness and heterogeneity of the concrete with three-grade circular aggregates are considered under different excitation frequencies. From Figure 9, a certain difference between the steady amplitudes of the embedded PZT sensor of the three samples under identical excitation frequency can be found. It means that the randomness and heterogeneity of the concrete core of the CFST member have a certain influence on the PZT sensor response of the mesoscale healthy substructure coupling models. Because the stress wave propagation from the PZT actuator to the PZT sensor is affected by the mechanical parameters of the aggregates and the mortar, the random distribution of the aggregates should lead to a certain difference in the PZT sensor response. However, for the studied three specimens with identical global mechanical performance, the response of the embedded PZT sensor is limited.

## 5. Wavelet Packet Energy of the Embedded PZT Sensor Measurement Considering Debonding under Sweep Frequency Excitation Signal

In this section, in order to distinguish the effect of the interface debonding defect of CFST members and that of the heterogeneity and randomness of the mesoscale substructures coupling models on the embedded PZT sensor measurement, the response of the embedded PZT sensor and its wavelet packet energy under a sweep frequency excitation signal is discussed in detail.

The sweep excitation signal employed to excite the surface-mounted PZT actuator is described in the following equations. The input voltage amplitude of the excitation signal is 10 V.

(16) $$V_{t}=V_{0}\sin\left(2\pi (f_{0}+\frac{f_{1}-f_{0}}{T}t\right)t)$$

where $V_{t}$, $V_{0}$ refer to excitation voltage signal and its amplitude, $f\left(t\right)$, $f_{0}$, $f_{1}$ are the frequency of signal at the time instant of t, initial frequency 10 kHz and final frequency 30 kHz in this study, and T is the time duration of the sweep frequency signal and takes the value of 1 ms in this study.

### 5.1. Effect of Interface Debonding Defect Compared with a Single Circular Aggregate Size

**Figure 10:**
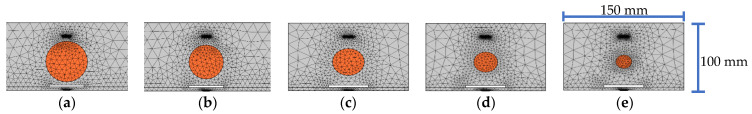
Mesoscale substructure with identical interface debonding defect and different circular aggregate sizes: (**a**) 60 mm; (**b**) 50 mm; (**c**) 40 mm; (**d**) 30 mm; (**e**) 20 mm.

In order to investigate the effect of the interface debonding defect on the response of embedded PZT sensors in the mesoscale substructure coupling models with different single circular aggregate sizes, five mesoscale substructures with an identical interface debonding defect, as shown in Figure 10, are established. The lateral position of the center of the PZT actuator and sensor, the aggregate and interface debonding defect are identical. The size of the interface debonding defect is 50 mm × 3 mm, and the diameters of the circular aggregate are identical to those shown in Figure 2.

**Figure 11:**
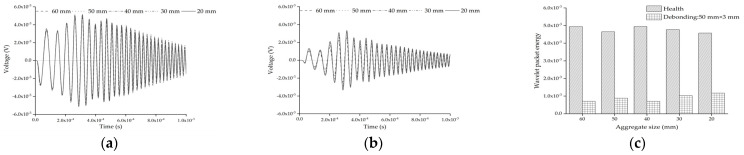
The PZT sensor measurement and the corresponding wavelet packet energy of substructures with different circular aggregate sizes: (**a**) health; (**b**) with debonding; (**c**) the comparison of wavelet packet energy value.

Figure 11a shows the comparison of the response of the embedded PZT sensor in the mesoscale substructure coupling models without the interface debonding defect under a sweep frequency excitation signal. It can be found that the difference between the embedded PZT responses is very limited, although the size of the single circular aggregate is different. Figure 11b shows the comparison of the responses of the embedded PZT sensor in the mesoscale substructure coupling models with the interface debonding defect under a sweep frequency excitation signal. Comparing the results shown in Figure 11a,b, it is clear that the response of the embedded PZT sensor in the healthy mesoscale CFST-PZT substructure coupling models is higher than that of the corresponding mesoscale substructure coupling models with the interface debonding defect. Figure 11c shows the comparison of the wavelet packet energies of the embedded PZT sensor response in the mesoscale substructure coupling models without and with the interface debonding defect. It is clear that the existence of the interface debonding defect leads to an obvious wavelet packet energy decrease even though the circular aggregate size is different. This result indicates that the interface debonding defect of the substructure of a CFST member leads to an obvious decrease in the response of the embedded PZT sensor, no matter what the circular aggregate size in the mesoscale substructure is. The circular aggregate size between the PZT actuator and sensor in the mesoscale substructure has less effect on the embedded PZT sensor measurement than its interface debonding defect.

### 5.2. Effect of Circular Aggregate Lateral Position Compared with Interface Debonding Defect

**Figure 12:**
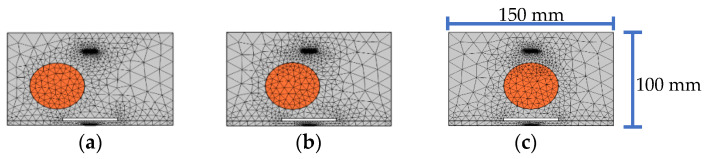
Mesoscale substructures with interface debonding defect and different aggregate lateral positions: (**a**) −30 mm; (**b**) −15 mm; (**c**) 0 mm.

In order to further investigate the effect of interface debonding defect on the response of embedded PZT sensors in the mesoscale substructure coupling models with different lateral positions of a single circular aggregate, three mesoscale substructure coupling models with an identical interface debonding defect and an identical circular aggregate at different lateral positions, as shown in Figure 12, are established. The diameter of the circular aggregate is 50 mm, and the size of the interface debonding defect is 50 mm × 3 mm.

**Figure 13:**
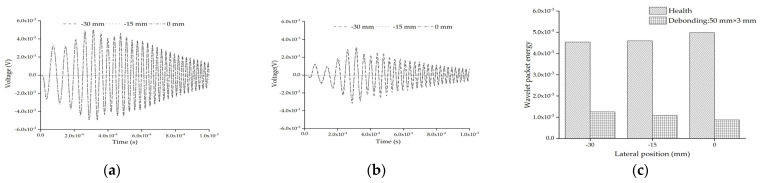
PZT sensor measurement and the corresponding wavelet packet energy under sweep frequency signal: (**a**) health; (**b**) with debonding; (**c**) the comparison of wavelet packet energy value.

Figure 13a shows the comparison of the response of the embedded PZT sensor in the mesoscale substructure coupling models without the interface debonding defect under the sweep frequency excitation signal. It can be found that the difference between the response of the embedded PZT responses is very limited, even though the aggregate lateral position is different. Figure 13b shows the comparison of the response of the embedded PZT sensor in the mesoscale substructure coupling models with an identical interface debonding defect under the sweep frequency excitation signal. Comparing the results shown in Figure 13a,b, it is clear that the response of the embedded PZT sensor in the healthy mesoscale substructure of the CFST-PZT coupling model is obviously higher than those of the mesoscale substructure coupling models with an interface debonding defect. Moreover, it can be seen from Figure 13c that the wavelet packet energy values of the embedded PZT sensor of the mesoscale substructure coupling models are less affected by the lateral position of aggregate. This finding is consistent with the results under the sinusoidal excitation signal. It is concluded that the effect of interface debonding defect on the wavelet packet energy values of the embedded PZT sensor measurement is dominant compared with that of the different lateral positions of the single circular aggregate.

### 5.3. Effect of Circular Aggregate Longitudinal Position Compared with Interface Debonding Defect

**Figure 14:**
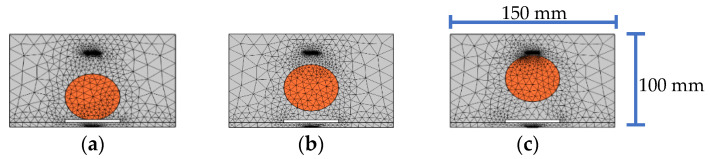
Samples of substructure with interface debonding defect and different longitudinal position of circular aggregate: (**a**) −100 mm; (**b**) 0 mm; (**c**) 100 mm.

**Figure 15:**
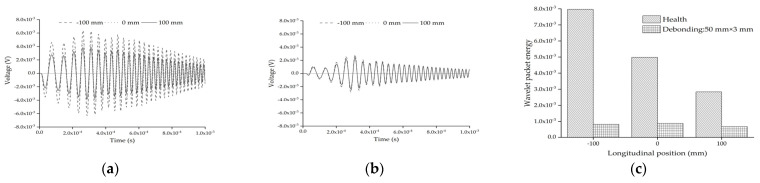
PZT sensor response and their corresponding wavelet packet energy of substructure without and with interface debonding defect considering different longitudinal positions of circular aggregate: (**a**) health; (**b**) with interface debonding; (**c**) the comparison of wavelet packet energy value.

Further investigation on the effect of the longitudinal position of the single circular aggregate on the embedded PZT sensor response compared with that of interface debonding defect is carried out. Figure 14 shows three mesoscale substructure coupling models with identical interface debonding and a single circular aggregate at different longitudinal positions, where the diameter of the circular aggregate is 50 mm and the size of the interface bonding defect is 50 mm × 3 mm. The embedded PZT sensor response and their corresponding wavelet packet energies of the substructure coupling models without and with the interface debonding defect considering different longitudinal positions of the circular aggregate are shown in Figure 15.

It can be seen from Figure 15a that the longitudinal position of the single circular aggregate in the healthy mesoscale substructure CFST-PZT coupling models has an obvious influence on the output voltage amplitude under the sweep frequency excitation. When the longitudinal position of the circular aggregate is closer to the embedded PZT sensor, the response of the PZT sensor is less than others. This trend is similar to that when sinusoidal excitation is employed. As shown in Figure 15b, the effect of the longitudinal position of the circular aggregate on the response of the embedded PZT sensor of the mesoscale substructure coupling models with interface debonding is very limited. Figure 15c shows the comparison of the wavelet packet energy of the embedded PZT sensor measurement in each healthy mesoscale substructure coupling model without and with the interface debonding defect. From Figure 15c, it is clear that the wavelet packet energy of the PZT sensor response of each healthy mesoscale substructure coupling model significantly decreases as the longitudinal distance between the aggregate and the PZT sensor decreases. However, the difference in wavelet packet energy values is minimal for the mesoscale substructure CFST-PZT coupling models with the interface debonding defect even though the aggregate longitudinal positions are different. Although the longitudinal position of the circular aggregate affects wavelet packet energy values on healthy substructure obviously, the effect of the interface debonding defect on the wavelet packet energy of the embedded PZT sensor response is dominant.

### 5.4. Effect of Aggregates Distribution of Mesoscale Substructures Compared with Interface Debonding Defect

**Figure 16:**
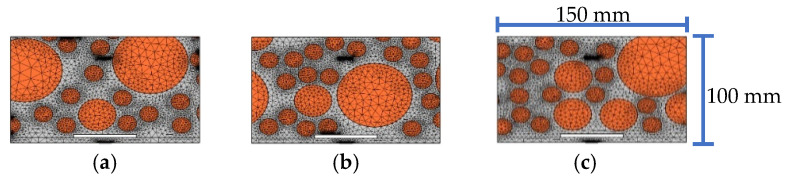
The samples of different aggregates distribution for substructures with debonding: (**a**) sample 1; (**b**) sample 2; (**c**) sample 3.

Without a loss of generality, the effect of an interface debonding defect on the embedded PZT sensor response in three different aggregates distributed substructure coupling models is further investigated. The three mesoscale substructures with their FEM mesh are shown in Figure 16, where the size of the debonding defect is 50 mm × 3 mm.

**Figure 17:**
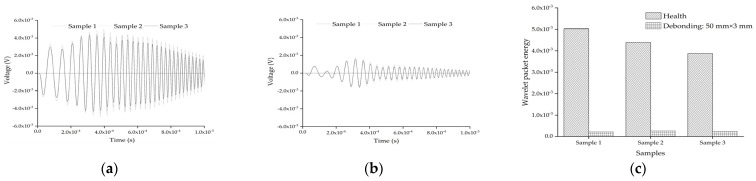
The PZT sensor response of substructures without and with interface debonding defect considering different distribution of aggregates: (**a**) health; (**b**) with interface debonding: 50 mm × 3 mm; (**c**) The comparison of wavelet packet energy value.

The response of the embedded PZT sensor of different mesoscale substructures without and with interface debonding defects is simulated, and the results are shown in Figure 17. From Figure 17a, it can be seen that the response of the embedded PZT sensor is affected by the local aggregates distributions in the mesoscale substructures between the PZT actuator and the PZT sensor when the distance between the PZT sensor and the PZT actuator is 80 mm. Compared with the healthy mesoscale substructure, it can be seen from Figure 17b that the aggregates distributions have little effect on the response of the embedded PZT sensors in the mesoscale substructure coupling models with interface debonding defects. In addition, from the wavelet packet energy analysis of the embedded PZT sensor measurement under the sweep excitation signal as shown in Figure 17c, it can be seen that although the aggregates distributions in the mesoscale substructure coupling models have a certain effect on the embedded PZT sensor response of the substructures without interface debonding, the interface debonding defect still plays a dominant role in affecting the PZT sensor response when the heterogeneity and randomness of concrete in CFST members is considered.

Compared with the results in the previous work about the response of the embedded PZT sensor in a whole mesoscale CFST-PZT coupling model with different aggregate distributions when the different interface debonding defects were considered [37], the results from the proposed mesoscale substructure coupling model described above can distinguish the effect of the mesoscale structure of concrete and those of the interface debonding defect on the response of PZT sensor in the mesoscale CFST-PZT coupling models. The findings from this study are consistent with those of the previous studies by the authors. It can be concluded that the stress wave simulation results by using the proposed mesoscale substructure coupling model are feasible.

### 5.5. Effect of Interface Debonding Defect Length When the Aggregates Distribution of Mesoscale Substructures Is Considered

**Figure 18:**
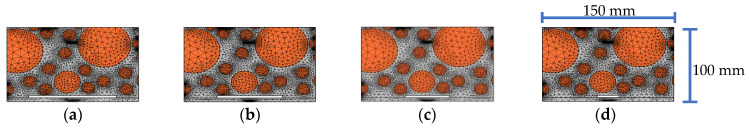
Substructures of CFST-PZT coupling members corresponding sample 1 with different debonding defect: (**a**) 100 mm × 3 mm; (**b**) 75 mm × 3 mm; (**c**) 50 mm × 3 mm; (**d**) 25 mm × 3 mm.

The effect of the interface debonding defect on the embedded PZT sensor response in different mesoscale substructures with different interface debonding defect lengths is further investigated here. Four mesoscale substructures with their FEM mesh are shown in Figure 18, where the thickness of the interface debonding defect is 50 mm, and the length of the debonding defect is 100 mm, 75 mm, 50 mm and 25 mm, respectively.

**Figure 19:**
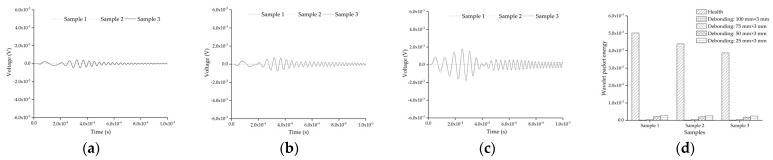
Samples of substructure of CFST-PZT coupling members with different debonding defects: (**a**) 100 mm × 3 mm; (**b**) 75 mm × 3 mm; (**c**) 25 mm × 3 mm; (**d**) the comparison of wavelet packet energy value.

The response of the embedded PZT sensor of each mesoscale substructure coupling model with the debonding length of 100 mm, 75 mm, and 25 mm is shown in Figure 19. The output signals of both healthy substructures and those with a debonding length of 50 mm have been shown in Figure 17a,b and are not shown here. It can be seen from Figure 17a,b and Figure 19a–c that as the length of the interface debonding defect decreases, the output voltage signal of the PZT sensor under sweep frequency excitation attenuates obviously. In Figure 19d, the wavelet packet energy corresponding to the response of the embedded PZT sensor in mesoscale substructures with different defect lengths are compared. The wavelet packet energy value of the embedded PZT sensor of the mesoscale substructure coupling model with debonding attenuates obviously with the increased interface debonding length. In addition, no matter what the length of the interface debonding defect is, the wavelet packet energy value of the mesoscale substructures with the interface debonding defect is always much lower than that of the healthy substructures. The effect of the debonding defect length on the response of the embedded PZT sensor in the mesoscale substructure coupling model is consistent with that of previous work about the response of the embedded PZT sensor in a whole coupling CFST model with different debonding defect lengths [37].

## 6. Concluding Remarks

In this study, a multi-physics simulation with mesoscale substructures of CFST-PZT coupling models composed of a surface-mounted PZT actuator, an embedded PZT sensor, aggregate, mortar and a steel tube was carried out, and the effect of the size, lateral and longitudinal positions of a single aggregate, the distribution of aggregates and the interface debonding defect on the response of the embedded PZT sensor was investigated in detail with the proposed mesoscale substructure coupling models. The output voltage response of the embedded PZT sensor of different mesoscale substructure coupling models without and with debonding under a continuous sinusoidal excitation signal or sweep frequency excitation signal was discussed. Based on the multi-physics mesoscale simulation results on each substructure, the following conclusions could be drawn:

(1) The steady output voltage amplitudes of the embedded PZT sensor of the mesoscale substructure coupling models showed that the size, lateral and longitudinal positions of a single aggregate and the aggregates distributions differently affected the response of embedded PZT sensor of the mesoscale substructures without the interface debonding defect under continuous sinusoidal excitation signal. The effect of the size, lateral position of a single aggregate and the aggregates distributions on the response of embedded PZT sensor of the mesoscale substructure without interface debonding was limited, but the aggregate longitudinal position had the most obvious influence.
(2) The effect of the size and position of a single aggregate and the distribution of aggregates on the response of the embedded PZT sensor of the mesoscale substructure coupling models with interface debonding defect was comparatively limited when compared with that of the mesoscale substructures without the interface debonding defect under sweep frequency excitation signal. The existence of interface debonding defect led to an obvious decrease in the output voltage amplitude of the embedded PZT sensor no matter what size and position of the single aggregate and distribution of aggregates were considered.
(3) The wavelet packet energy of the embedded PZT sensors is also dominantly affected by the interface debonding defect rather than the mesoscale structure of the concrete core of the substructure coupling models with different single aggregate sizes, positions and aggregates distribution. Additionally, the length of the interface debonding defect had an obvious effect on the wavelet packet energy of the embedded PZT sensor in the mesoscale substructure coupling models and its wavelet packet energy of the models with the interface debonding defect was always much lower than that of the healthy substructure.

In this study, the distance between the PZT actuator and the PZT sensor was constant. In order to demonstrate the effect of the measuring distance between the PZT actuator and sensor on the embedded PZT sensor of the mesoscale substructures without interface debonding defect, further investigation on the response of the output voltage signal of the PZT sensor with different measuring distances could be carried out in future. Moreover, the mesoscale structure of concrete with aggregate shapes other than the circular shape can also be considered further.

## Figures and Tables

**Table 1:** Definition of mesoscale parameters of substructures of CFST-PZT.

No.	Aggregate Size (mm)	Lateral Position (mm)	Longitudinal Position (mm)
1	60	−30	−20
2	50	−15	0
3	40	0	20
4	30	/	/
5	20	/	/

**Table 2:** Material properties.

Material	Elastic Modulus (GPa)	Poisson’s Ratio	Density (kg/m^3^)
Steel	200	0.33	7850
Aggregate	55.5	0.16	2700
Mortar	26	0.22	2400

**Table 3:** Number of elements corresponding to different frequencies for the mesoscale coupling substructure model shown in Figure 1b.

Frequency	Number of Elements
10 kHz	1376
20 kHz	1417
30 kHz	1860

## Data Availability

Data are contained within in the article.
